# Terrestrial Bird Migration and West Nile Virus Circulation, United States

**DOI:** 10.3201/eid2412.180382

**Published:** 2018-12

**Authors:** Daniele Swetnam, Steven G. Widen, Thomas G. Wood, Martin Reyna, Lauren Wilkerson, Mustapha Debboun, Dreda A. Symonds, Daniel G. Mead, Barry J. Beaty, Hilda Guzman, Robert B. Tesh, Alan D.T. Barrett

**Affiliations:** University of California at Davis, Davis, California, USA (D. Swetnam);; University of Texas Medical Branch, Galveston, Texas, USA (D. Swetnam, S.G. Widen, T.G. Wood, H. Guzman, R.B. Tesh, A.D.T. Barrett);; Harris County Public Health, Houston, Texas, USA (M. Reyna, L. Wilkerson, M. Debboun);; Chesapeake Mosquito Control Commission, Chesapeake, Virginia, USA (D.A. Symonds);; University of Georgia, Athens, Georgia, USA (D.G. Mead);; Colorado State University, Fort Collins, Colorado, USA (B.J. Beaty)

**Keywords:** West Nile virus, bird migration, phylogeography, emerging pathogens, viruses, terrestrial birds, United States, vector-borne infections, zoonoses

## Abstract

Host migration and emerging pathogens are strongly associated, especially with regard to zoonotic diseases. West Nile virus (WNV), a mosquitoborne pathogen capable of causing severe, sometimes fatal, neuroinvasive disease in humans, is maintained in highly mobile avian hosts. Using phylogeographic approaches, we investigated the relationship between WNV circulation in the United States and the flight paths of terrestrial birds. We demonstrated southward migration of WNV in the eastern flyway and northward migration in the central flyway, which is consistent with the looped flight paths of many terrestrial birds. We also identified 3 optimal locations for targeted WNV surveillance campaigns in the United States—Illinois, New York, and Texas. These results illustrate the value of multidisciplinary approaches to surveillance of infectious diseases, especially zoonotic diseases.

West Nile virus (WNV) is a mosquitoborne virus that can cause severe and even fatal disease in humans. After WNV introduction into New York, NY, USA, its geographic range expanded quickly, reaching the West Coast in 2003. Previous studies have shown that the spread of WNV occurred faster than could be explained by contiguous diffusion (*1*–*4*) and demonstrated that its expansion occurred heterogeneously, consisting of contiguous diffusion and long distance translocations (*2*,*5*). Since then, phylogeographic studies have reported frequent mixing of WNV strains from local and distant locations. The most notable exception is California**,** where several genetic studies have shown limited movement into and out of the state (*6**,**7*).

The rapid expansion of WNV in the United States probably cannot be attributed to the movement of humans because humans are dead-end hosts. However, in nature, WNV is maintained in an enzootic transmission cycle involving mosquito vectors and highly mobile avian reservoirs. *Hyalomma marginatum* ticks have also been implicated in the transmission of WNV (*8*).

Although evidence of WNV infection has been identified in many species of birds, deaths and disease among birds vary greatly, ranging from asymptomatic to fatal infections; peak viremia potentially reaches >10^12^ PFU/mL (*9*). WNV RNA has been detected in bird spleen and kidneys as long as 36 weeks after infection (*10*) and in brains of *Nestor notabilis* kea up to 72 months after infection (*11*).

Although phylogenetic evidence of geographic clustering by location is limited, a recent study reported that WNV isolates clustered according to avian flyway (*12*). Because birds are the primary reservoirs for WNV, this finding was not surprising, but it is relevant because bird migration has also been implicated in the movement of influenza A virus (*13*), *Borrelia burgdorferi* (Lyme disease agent) (*14*), other pathogenic organisms (*1*), and even invasive invertebrate organisms (*15*). Several serologic studies (e.g., ELISA, plaque reduction neutralization test) have been used to determine the direction of WNV movement within the Atlantic, Mississippi, and Pacific flyways and demonstrated WNV in birds migrating southward, whereas evidence of the virus in birds during northward migration is limited (*16*,*17*).

Studies of virus movement associated with avian hosts in the United States have concentrated on the migration of waterfowl and excluded terrestrial birds, largely because the migratory patterns of waterfowl have been thoroughly characterized by banding studies. However, passerine birds, the primary reservoir for WNV, are terrestrial birds, not waterfowl. Terrestrial birds and waterfowl fly along similar but distinct flyways. Although waterfowl follow regular paths bounded by mountains and rivers, terrestrial birds often follow looped routes that enable them to maximize tail winds, avoid head winds, and correlate with seasonal fluctuations in food availability (*18*,*19*). Although looped migration paths have been described for several species of birds (*Selasphorus rufus* hummingbirds [*20*], *Circus aeruginosus* western marsh harriers [*21*], *Falco eleonorae* Eleonora’s falcons [*22*], *Cuculus canorus* common cuckoos [*23*]), the general flyways of terrestrial birds have been inadequately studied. However, in 2014, La Sorte et al. provided a general description of terrestrial bird flyways in North America (*18*). They defined 3 flyways: the single distinct Western flyway and 2 overlapping flyways, the Central and Eastern flyways. A similar 3-flyways system (Pacific, Central, and Atlantic flyways) has been described for waterfowl (*24*); however, most studies have relied on the more common 4-flyways system (Pacific, Central, Mississippi, and Atlantic flyways). In this study, we used phylogeographic approaches to investigate the relationship between WNV circulation in the United States and the flight paths of terrestrial birds. 

## Methods

### Generation of Alignments

We identified all unique sequences of natural and laboratory WNV strains by using the Virus Variation Resource (*25*). Virus sequences meeting the following criteria were included in this study: 1) the nucleotide sequence spanned the complete open reading frame, 2) the sequence was derived from natural isolates and not laboratory strains, 3) the sequence was unique (i.e., all sequences differed by >1 nt), and 4) the sequence contained no degenerate nucleotides. All sequences were manually aligned in BioEdit version 7.1.3 (http://www.mbio.ncsu.edu/BioEdit/bioedit.html) or MEGA7 (https://www.megasoftware.net/), and noncoding regions were removed when necessary (i.e., the open reading frame was used for analyses).

### Isolation of Viral RNA and Next-Generation Sequencing

We obtained additional WNV isolates from the World Reference Center for Emerging Viruses and Arboviruses at the University of Texas Medical Branch at Galveston (Galveston, TX, USA) (*26*). Isolates were originally collected from Virginia, Georgia, Texas, and Colorado. We extracted viral RNA from the supernatant of infected Vero cells by using a QIAamp Viral RNA Mini Kit (QIAGEN, Germantown, MD, USA) according to the manufacturer’s instructions. We generated libraries with a TruSeq RNA version 2 kit (Illumina, San Diego, CA, USA) and samples sequenced by the University of Texas Medical Branch at Galveston Next Generation Sequencing Core on an Illumina 1500 Hi-Seq platform. Adaptor sequences and poor quality reads (Q score <20) were removed with Trimmomatic (*27*). Reads were aligned with Bowtie2 (*28*) under the sensitive local parameter against the prototypical strain of WNV (NY99 flamingo 382–99, GenBank accession no. AF196835). Consensus sequences were generated by using SAMtools (*29*).

### Phylogeny

To evaluate temporal structure, we generated a time-naive phylogeny (i.e., a maximum-likelihood phylogeny) to enable determination of the patristic distance between all isolates on the phylogeny. We generated maximum-likelihood trees with RAxML-HPC Black Box on Cyberinfrastructure for Phylogenetic Research version 3.3 (*30*) and determined automatic halting by bootstrapping. We determined the root-to-tip distance, which is a phylogenetic measure of genetic distance, for each isolate of the maximum-likelihood phylogenies by using TempEst (formerly Path-o-gen) (*31*). We evaluated the correlation (Pearson method) between root-to-tip distance and collection date in R (https://www.r-project.org/).

We used a Bayesian Markov chain Monte Carlo (MCMC) approach to infer phylogeographic relationships and selected the most appropriate phylogenetic model by using standard path sampling and stepping-stone approaches. XML files were generated in BEAUti version 1.8.3 or 1.8.4 and run with BEAST version 1.8.4 (*32*) on Cyberinfrastructure for Phylogenetic Research (*30*). We used the GTR+Γ+I (general time reversible with gamma rate distribution and invariable sites) model to infer nucleotide substitution frequencies, an uncorrelated lognormal clock model to infer the mutation rate, and a Bayesian Skyline tree prior to model changes in population size. The evolution rate mean was restricted to 10^−4^ through 9 × 10^−4^ substitutions/site/year, consistent with previously reported rates for WNV evolution (*7*,*33*).

We ran trees with an MCMC length of 100 million and sampled every 5,000 steps. Log files were reviewed in Tracer (http://tree.bio.ed.ac.uk/software/tracer/) to determine burn-in, which ranged from 5% through 10%. We ran multiple independent MCMC chains until effective sample size values exceeded 200. Log and tree files were combined in LogCombiner version 1.8, and a maximum clade credibility tree was generated in TreeAnnotator (*32*). Locations were inferred by using ancestral state reconstruction with an asymmetric discrete trait substitution model (*34*).

### Analysis of Migration

After the XML files were generated in BEAUti, we manually edited them to enable counting of all Markov jumps (MJ) (which described the relative magnitude of migration out of source locations and into sink locations) for 2001 through 2009 (*35*). This method for evaluating migration, first described by Minin and Suchard (*35*), has been used to characterize migration of several major pathogens including rabies virus (*36*), dengue virus (*37*), HIV (*38*), influenza virus (*39*,*40*), and Rift Valley fever virus (*41*).

As expected for an emerging zoonotic disease, the annual West Nile neurologic disease (WNND) incidence and sample collection efforts varied dramatically among states over time, adding substantial complexity to the model. To mitigate the effects of inconsistent sampling and to confirm the observed results, we applied a stricter inclusion criterion to confirm the results obtained by using the full dataset. The sequences were randomly down-sampled such that the number of sequences used correlated (p<0.05 by Pearson method) with the incidence of WNND reported to the Centers for Disease Control and Prevention (CDC) in a particular year (the most accurate record of relative WNV activity). Our analysis ensured that the dataset was representative of the WNV activity of each region in a particular year.

We calculated incidence by using the number of WNND cases reported to CDC from each state during each year and dividing that number by the estimated population of each state. The population estimates were obtained from the Time Series of Intercensal State Population Estimates available at the Population Division of the US Census Bureau (*42*). States with insufficient sequences to represent the WNND incidence were excluded. Down-sampling was undertaken in at least duplicate to ensure that reduction in sample size and diversity did not remove important relationships.

## Results

### Sequence Collection

All previously published sequences of natural WNV isolates collected in the United States were obtained from GenBank on January 1, 2016. The number of WNV sequences varied substantially over time and among locations, which presented statistical challenges. In particular, although GenBank has >900 WNV open reading frames, most come from a few states where laboratories were actively involved in WNV surveillance and research: California, New York, and Texas. The ability to compare multiple isolates over multiple years was critical to the analysis. Only a few states had sufficient numbers of WNV sequences available in GenBank to enable analysis for multiple consecutive years: New York, Connecticut, Illinois, North Dakota, South Dakota, Texas, and California.

To mitigate the influence of sampling bias, we obtained additional WNV isolates from the World Reference Center for Emerging Viruses and Arboviruses for 3 states and sequences to support the analysis: Virginia (n = 39), Georgia (n = 20), and Colorado (n = 31) (Table 1). Given that previous studies have demonstrated limited WNV movement into or out of California (*6*,*7*), we did not include isolates from California in the analysis. Similarly, because of the proximity of New York and Connecticut, we chose New York to represent WNV in the Northeast because Connecticut is a small state. Last, to ensure that each location was represented across a similar time frame, we included only isolates collected during 2001–2009 in the Bayesian phylogeny and migration analysis. Table 2 shows the states and availability of yearly isolates.

**Table 1:** Summary of isolates sequenced in study of terrestrial bird migration and West Nile virus circulation, United States

Isolate	Phylogeny code	GenBank accession no.	State	Year
Laco_3008	CO03C	MH170226	CO	2003
AIDL-M-015	CO03D	MH170228	CO	2003
LACO-3041	CO03E	MH170231	CO	2003
Laco_3038	CO03F	MH170234	CO	2003
AIDL-M-012	CO03G	MH170237	CO	2003
Laco_3020	CO03H	MH170238	CO	2003
Laco_3022	CO03I	MH170254	CO	2003
laco_3030	CO03J	MH170256	CO	2003
CO1862	CO04E	MH170227	CO	2004
CO_2572	CO04F	MH170246	CO	2004
DB_4218	CO04G	MH170248	CO	2004
DB_4217	CO04H	MH170233	CO	2004
CO_06–7390	CO06A	MH170229	CO	2006
CO_06–608	CO06B	MH170232	CO	2006
CO_06–10725	CO06C	MH170235	CO	2006
CO_07–11032	CO06D	MH170236	CO	2006
CO_06–10723	CO06E	MH170239	CO	2006
CO_06–584	CO06F	MH170243	CO	2006
CO_06–10716	CO06G	MH170249	CO	2006
CO_07–8779	CO07C	MH170230	CO	2007
CO_07–10970	CO07D	MH170241	CO	2007
GT_02566	CO07E	MH170242	CO	2007
CO_07–8778	CO07F	MH170244	CO	2007
CO_07–11027	CO07G	MH170251	CO	2007
CO_07–9340	CO07H	MH170252	CO	2007
CO_08–13382	CO08A	MH170240	CO	2008
CO_08–13386	CO08B	MH170245	CO	2008
CO-13363	CO08C	MH170247	CO	2008
CO_08–13401	CO08D	MH170250	CO	2008
CO_08–13787	CO08E	MH170253	CO	2008
CO_08–13410	CO08F	MH170255	CO	2008
DES_566–01	GA01C	MH170263	GA	2001
DES_107–01	GA01D	MH170273	GA	2001
DES_1476–01	GA01E	MH170276	GA	2001
DES_1191–02	GA02C	MH170274	GA	2002
DES_160–02	GA02D	MH170275	GA	2002
DES_1201–02	GA02E	MH170264	GA	2002
GA_04–230	GA04A	MH170270	GA	2004
GA_Chc_04–1485	GA04B	MH170265	GA	2004
GA_05–179	GA05A	MH170269	GA	2005
GA_lwn_50_4936	GA05B	MH170257	GA	2005
M07–069	GA07A	MH170272	GA	2007
M07–087	GA07B	MH170258	GA	2007
M07–086	GA07C	MH170266	GA	2007
DES_07–53	GA07D	MH170267	GA	2007
DES_07–62	GA07E	MH170268	GA	2007
DKB_08–0403	GA08A	MH170261	GA	2008
DBK_08–0491	GA08B	MH170271	GA	2008
FNT_09–199	GA09A	MH170259	GA	2009
Lwn_09–846	GA09B	MH170262	GA	2009
FNT_09–144	GA09C	MH170260	GA	2009
VA_AV_321–00	VA00A	MH166882	VA	2000
VA_AV_593	VA00B	MH166904	VA	2000
VA_AV_380	VA00C	MH166903	VA	2000
VA_AV_573–00	VA00D	MH166901	VA	2000
VA_TC_2535–01	VA01A	MH166886	VA	2001
VA_B_037–02	VA02A	MH166883	VA	2002
VA_BD_37	VA02B	MH166905	VA	2002
VA_TC_1500	VA02C	MH166887	VA	2002
VA_TC_1500–02	VA02D	MH166899	VA	2002
VA_TC_2076	VA02E	MH166911	VA	2002
VA_TC_2147	VA02F	MH166915	VA	2002
VA_TC_2790–03	VA03C	MH166906	VA	2003
VA_TC_4043	VA03D	MH166900	VA	2003
VA_TC_3278	VA03E	MH166912	VA	2003
VA_1909–04	VA04A	MH166884	VA	2004
VA_TC_1597	VA04B	MH166919	VA	2004
VA_TC_1155	VA04C	MH166913	VA	2004
VA_TC_1272	VA04D	MH166917	VA	2004
VA_P_3321–05	VA05A	MH166888	VA	2005
VA_P_4209	VA05B	MH166895	VA	2005
VA_SN_3082–05	VA05C	MH166908	VA	2005
VA_P_4485–06	VA06A	MH166889	VA	2006
VA_P_4770–06	VA06B	MH166907	VA	2006
VA_SP_5645–06	VA06C	MH166890	VA	2006
VA_TC_4177	VA06D	MH166910	VA	2006
VA_1660	VA07A	MH166891	VA	2007
VA_2327	VA07B	MH166894	VA	2007
VA_TC_1368–08	VA08A	MH166898	VA	2008
VA_SP_1202–08	VA08B	MH166892	VA	2008
VA_TC_2045–08	VA08C	MH166921	VA	2008
VA_TC_1732–08	VA08D	MH166918	VA	2008
VA_3920	VA09A	MH166885	VA	2009
VA_SN_3222–09	VA09B	MH166909	VA	2009
VA_SN_5859–09	VA09C	MH166896	VA	2009
VA_TC_1732–09	VA09D	MH166920	VA	2009
VA_SN_4826–09	VA09E	MH166914	VA	2009
VA_2191	VA10A	MH166902	VA	2010
VA_TC_1117–10	VA10B	MH166897	VA	2010
VA_TC_2020–10	VA10C	MH166893	VA	2010
VA_TC_1184–10	VA10D	MH166916	VA	2010

**Table 2:** Years in which West Nile virus sequences were available in study of terrestrial bird migration and West Nile virus circulation, United States*

Location	1999	2000	2001	2002	2003	2004	2005	2006	2007	2008	2009	2010	2011	2012	2013	2014
NY	x		x	x	x	x	x		x	x			x			
VA		x	x	x	x	x	x	x	x	x	x	x				
GA			x	x		x	x		x	x	x					
IL				x	x	x	x	x	x							
TX				x	x		x	x	x		x	x	x	x	x	x
CO				x	x	x		x	x	x	x					
ND				x	x	x	x	x		x	x					
SD					x	x	x	x	x	x	x					

*x, available; blank cells, not available.

### Model Selection

**Figure 1:**
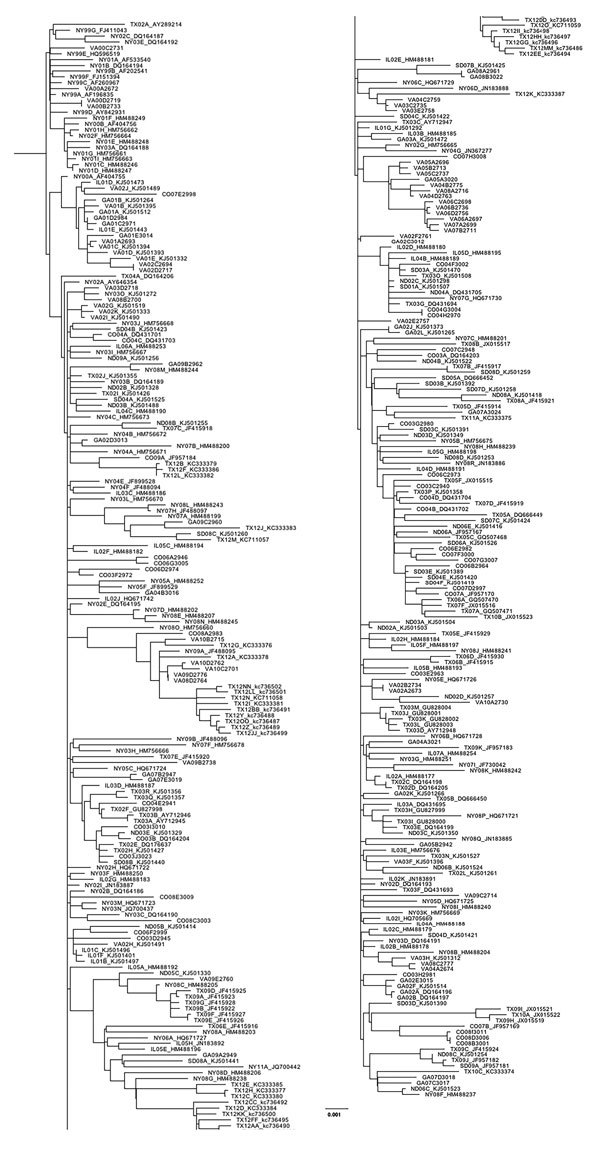
Maximum-likelihood phylogeny generated with all West Nile virus sequences from New York, Virginia, Georgia, Illinois, North Dakota, South Dakota, Texas, and Colorado (n = 379) in study of terrestrial bird migration and West Nile virus circulation, United States. Sequence names include the 2-letter state abbreviation to indicate the origin of isolation, followed by the year. Multiple isolates collected from the same state within the same year are differentiated by letter. GenBank accession numbers are provided for all taxa that were not sequenced in this study. Scale bar indicates nucleotide substitutions per site.

**Figure 2:**
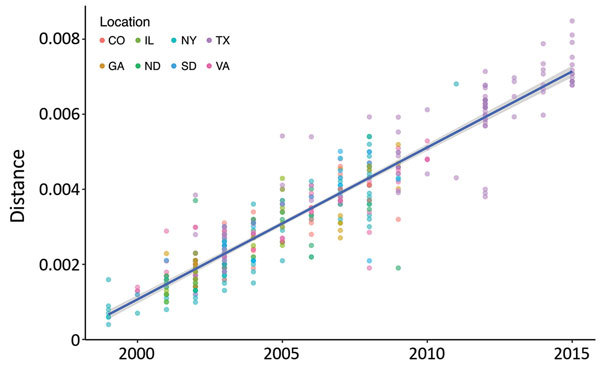
Analysis of correlation between virus isolation date and genetic diversity in study of terrestrial bird migration and West Nile virus circulation, United States. Root-to-tip distances of all sequences were determined for each isolate by using the maximum-likelihood tree shown in Figure 1 (https://wwwnc.cdc.gov/EID/article/24/12/18-0382-F1.htm) and plotted against the year. Dots are colored by location of isolation. The correlation between the root-to-tip distance and year of isolation was determined with linear regression shown in blue. 95% CIs are shown in gray. The equation of the linear regression line was used to estimate the year of the most recent common ancestor (MRCA) and the mutation rate (m): y = mx + MRCA.

We compared 203 nucleotide substitution models by using the Bayesian and Akaike Information Criteria in JModelTest2 (https://github/com/ddariba/jmodeltest2) and found the GTR+Γ+I model to be the most appropriate. For assessing temporal signature, we used a maximum-likelihood tree with sequences of WNV strains from New York, Virginia, Georgia, Illinois, North Dakota, South Dakota, Texas, and Colorado (n = 379) (Figure 1). We identified a statistically significant positive correlation (r = 0.93, 95% highest posterior density [HPD] = 0.92–0.94; p<2.2 × 10^−16^) between the root-to-tip distance and the date of isolation in Temp-Est (formerly known as Path-O-gen) (Figure 2). The mutation rate was estimated to be 4.05 × 10^−4^ substitutions/site/year, and the most recent common ancestor (MRCA) was in 1997. Together these results indicated a strong temporal signal in the dataset. Finally, we evaluated Bayesian tree priors (skyride, skygrid, and skyline) and uncorrelated clock models (lognormal and exponential) by using path-sampling and stepping-stone approaches. The uncorrelated lognormal clock model with the Bayesian skyline tree prior was the most appropriate.

### Phylogeographic Analysis for the United States

**Figure 3:**
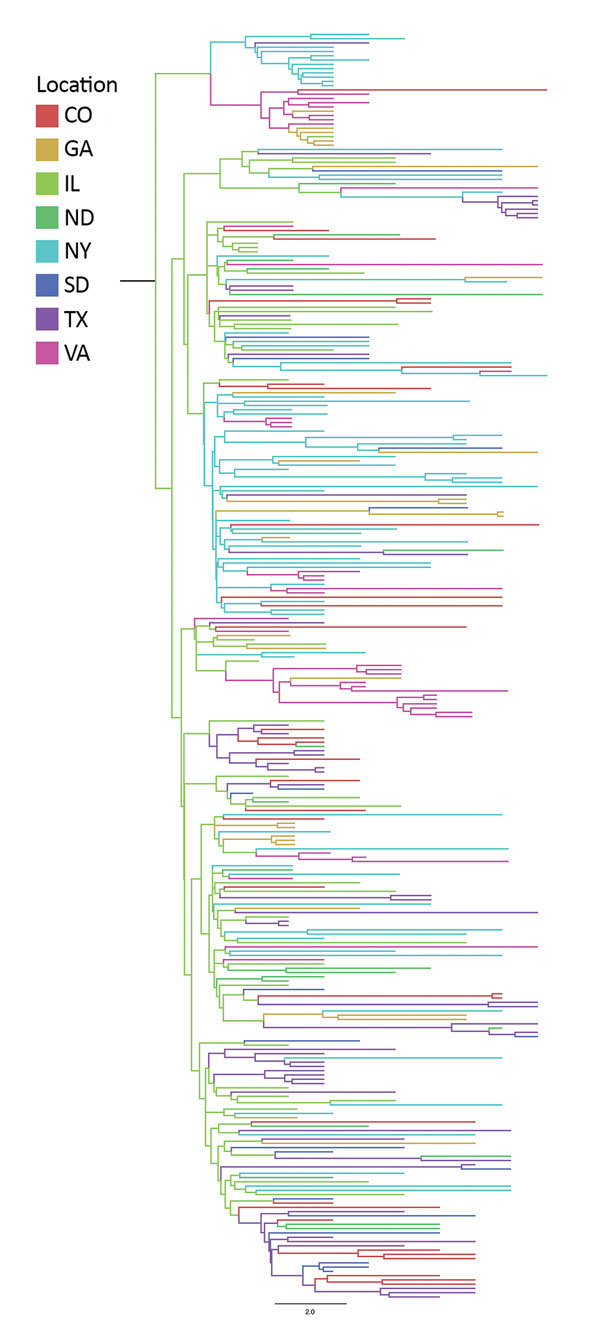
Bayesian phylogeny of West Nile virus isolates collected in representative regions along the Eastern and Central flyways between 2001 and 2009, United States. Maximum-clade credibility tree was obtained by using a Bayesian approach. The location of each isolate and the inferred location of each ancestor are depicted by color.

Analysis of all WNV sequences collected from New York, Virginia, Georgia, Illinois, North Dakota, South Dakota, Texas, and Colorado during 2001–2009 provided estimates of the introduction date of the MRCA and mean evolution rate that were consistent with the estimates of the root-to-tip distance analysis (Table 3; Figure 3). The date of MRCA introduction was estimated as 1997, and the average evolution rate was 3.92 × 10^−4^ substitutions/site/year.

**Table 3:** Statistical support for phylogeny in study of terrestrial bird migration and West Nile virus circulation, United States*

Variable	Mean	ESS	95% HPD interval
Posterior	−49722.50	1370	−49803.21 to −49640.55
Prior	−3987.09	1110	−4051.92 to −3916.56
Likelihood	−45735.41	2179	−45780.00 to −45691.43
MRCA	11.92	3119	10.82 to 13.08
UCLD.mean	3.92 × 10^−4^	1604	3.55 × 10^−4^ to 4.49 × 10^−4^

*ESS, effective sample size; HPD, highest posterior density; MRCA, most recent common ancestor (years before 2009); UCLD.mean, evolution rate inferred with an uncorrelated clock model with lognormal distribution.

We used MJ between reconstructed ancestral states to estimate the magnitude of relative migration out of, or into, each of the 8 regions (Table 4, 5). Frequent migration (>2 MJ) was detected from Illinois to Colorado (8.38 MJ), Illinois to Georgia (8.23 MJ), Illinois to North Dakota (10.43 MJ), Illinois to New York (29.97 MJ), Illinois to South Dakota (6.69 MJ), Illinois to Texas (22.87 MJ), Illinois to Virginia (11.45 MJ), New York to Colorado (4.36 MJ), New York to Georgia (7.04 MJ), New York to South Dakota (2.18 MJ), New York to Texas (4.56 MJ), New York to Virginia (4.24 MJ), Texas to Colorado (9.78 MJ), Texas to North Dakota (5.18 MJ), Texas to South Dakota (7.56 MJ), and Virginia to Georgia (3.62 MJ).

**Table 4:** Source and sink analysis in study of terrestrial bird migration and West Nile virus circulation, United States*

Source	Sink	Markov jumps, mean	ESS	95% HPD interval
CO	ND	1.368	8603	0–3
GA	IL	1.31	31284	1–3
IL	CO	8.376	3311	1–14
IL	GA	8.226	3046	3–13
IL	ND	10.43	15423	6–14
IL	NY	29.965	1355	20–40
IL	SD	6.691	3635	1–11
IL	TX	22.872	4545	14–30
IL	VA	11.449	1631	6–16
NY	CO	4.362	2143	0–8
NY	GA	7.039	2170	2–11
NY	IL	1.012	3023	0–4
NY	SD	2.177	2740	0–5
NY	TX	4.564	2149	0–9
NY	VA	4.24	1171	0–8
SD	CO	1.328	3964	0–5
TX	CO	9.775	6039	3–16
TX	ND	5.177	7617	2–9
TX	NY	1.606	9410	0–4
TX	SD	7.557	5113	2–12
VA	CO	1.16	9807	0–3
VA	GA	3.616	14054	2–6

*Mean number of Markov jjumps detected between each source (origin) and sink (destination) location indicates the minimum number of migration events observed from each source to each sink. Only Markov jumps >2 are shown. A summary of all Markov jumps is shown in Table 5. *ESS, effective sample size; HPD, highest posterior density.

**Table 5:** Markov jump analysis results from study of terrestrial bird migration and West Nile virus circulation, United States*

Source	Sink	Markov jumps	ESS	95% HPD interval
CO	GA	0.164	15683	0–1
CO	IL	0.111	33920	0–1
CO	ND	1.368	8603	0–3
CO	NY	0.261	16508	0–1
CO	SD	0.486	8222	0–2
CO	TX	0.337	5909	0–2
CO	VA	0.165	24157	0–1
GA	CO	0.314	13738	0–2
GA	IL	1.31	31284	1–3
GA	ND	0.122	26193	0–1
GA	NY	0.821	6736	0–3
GA	SD	0.719	8787	0–2
GA	TX	0.313	11570	0–2
GA	VA	0.334	10761	0–2
IL	CO	8.376	3311	1–14
IL	GA	8.226	3046	3–13
IL	ND	10.43	15423	6–14
IL	NY	29.965	1355	20–40
IL	SD	6.691	3635	1–11
IL	TX	22.872	4545	14–30
IL	VA	11.449	1631	6–16
ND	CO	0.618	8832	0–3
ND	GA	0.147	23908	0–1
ND	IL	0.144	25515	0–1
ND	NY	0.288	12855	0–2
ND	SD	0.183	20239	0–1
ND	TX	0.521	9813	0–2
ND	VA	0.255	15630	0–1
NY	CO	4.362	2143	0–8
NY	GA	7.039	2170	2–11
NY	IL	1.012	3023	0–4
NY	ND	0.448	7434	0–2
NY	SD	2.177	2740	0–5
NY	TX	4.564	2149	0–9
NY	VA	4.24	1171	0–8
SD	CO	1.328	3964	0–5
SD	GA	0.567	10905	0–2
SD	IL	0.143	26368	0–1
SD	ND	0.301	8901	0–2
SD	NY	0.236	19732	0–1
SD	TX	0.93	3517	0–4
SD	VA	0.116	32792	0–1
TX	CO	9.775	6039	3–16
TX	GA	0.669	6777	0–3
TX	IL	0.441	10249	0–2
TX	ND	5.177	7617	2–9
TX	NY	1.606	9410	0–4
TX	SD	7.557	5113	2–12
TX	VA	0.296	13706	0–2
VA	CO	1.16	9807	0–3
VA	GA	3.616	14054	2–6
VA	IL	0.261	14878	0–1
VA	ND	0.194	20523	0–1
VA	NY	0.96	4048	0–3
VA	SD	0.176	23197	0–1
VA	TX	0.247	15068	0–1

*ESS, effective sample size; HPD, highest posterior density.

**Figure 4:**
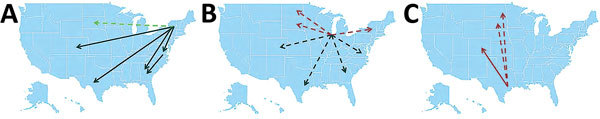
Summary of source/sink analysis in study of terrestrial bird migration and West Nile virus circulation, United States. Minimum number of migration events detected from A) the Eastern flyway, B) Illinois, and C) the Central flyway. Only events that occurred at least twice are depicted. Red arrows, northward migration; black arrows, southward migration; green arrow, lateral migration; dotted arrows, migration that could not be confirmed by incident-controlled downsampling because of an insufficient number of sequences.

Overall, 3 major sources of WNV circulation (New York, Illinois, and Texas) seemed to be the origin of 88.5% of the total migration events observed (Table 4; Figure 4). Southward and westward movements were detected along the East Coast, but only northward movement was observed within the central United States. A notable exception was observed in Illinois, where evidence of WNV movement in all directions was demonstrated.

### Incidence-Controlled Phylogeny

To mitigate the effects of inconsistent sampling, we applied a stricter inclusion criterion to ensure that the dataset was representative of WNV activity in each region in a particular year. In this approach, the sequences were randomly down-sampled by using the sample command in R, such that the number of sequences was proportional to the incidence of WNND reported to CDC (Table 6). Illinois, North Dakota, and South Dakota were not included in the down-sampled datasets because there were insufficient sequences to represent WNND incidence in these states. To ensure that reduction in sample size and diversity did not remove important relationships, the down-sampling was independently performed twice.

**Table 6:** Incidence-controlled down-sampling strategy used in study of of terrestrial bird migration and WNV circulation, United States*

Location, year	WNV incidence	Sequences available	Sequences used
GA			
2001	7.16 × 10^−7^	5	3
2002	3.29 × 10^−6^	11	6
2003	3.13 × 10^−6^	1	1
2004	1.60 × 10^−6^	2	2
2005	1.01 × 10^−6^	1	1
2006	2.18 × 10^−7^	0	0
2007	2.46 × 10^−6^	5	5
2008	4.21 × 10^−7^	3	3
2009	4.16 × 10^−7^	3	3
NY			
2001	6.81 × 10^−7^	10	3
2002	3.55 × 10^−6^	10	6
2003	2.97 × 10^−6^	15	5
2004	3.65 × 10^−7^	10	3
2005	1.57 × 10^−6^	7	4
2006	8.37 × 10^−7^	5	3
2007	8.36 × 10^−7^	9	3
2008	1.67 × 10^−6^	18	4
2009	3.11 × 10^−7^	2	2
VA			
2001	0	6	2
2002	2.20 × 10^−6^	10	5
2003	2.58× 10^−6^	6	5
2004	6.69 × 10^−7^	4	3
2005	0	3	2
2006	0	4	2
2007	3.87 × 10^−7^	2	2
2008	0	4	2
2009	6.31 × 10^−7^	5	3

*The numbers of West Nile virus sequences available and of sequences used in the down-sampled dataset are summarized. WNV, West Nile virus.

**Figure 5:**
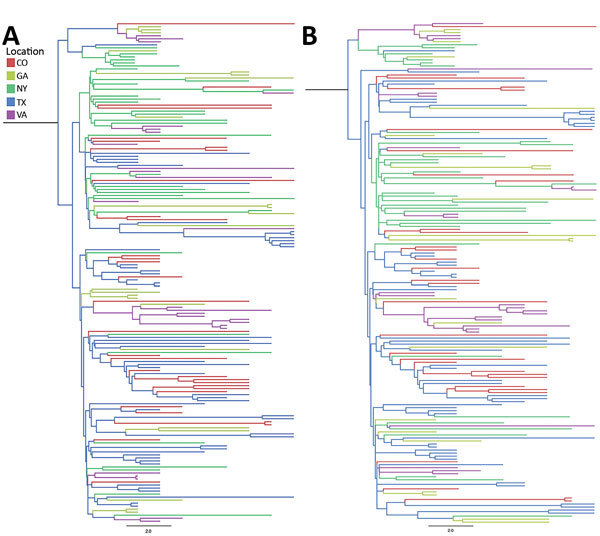
Incidence-controlled phylogeny of Eastern and Central flyways, United States. Sequences were down-sampled such that the number of sequences was proportional to the annual incidence of West Nile neurologic disease incidence for each location between 2001 and 2009. Down-sampling was undertaken twice (A and B) to ensure that the reduction in sequences did not result in a substantial loss of diversity. Illinois, North Dakota, and South Dakota were not included in the incidence-control analysis because too few sequences were available to support down-sampling. Bayesian approaches were used to generate maximum-clade credibility trees. Scale bars indicate nucleotide substitutions per site.

**Figure 6:**
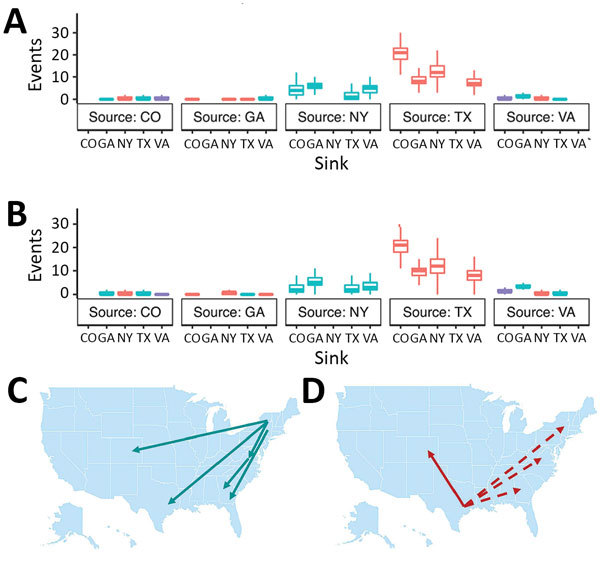
Summary of Markov jump analysis performed on the incident-controlled phylogeny. A, B) The results of the Markov jump analysis for each down-sampled dataset are summarized as box plots. Box tops indicate third quartiles, box bottoms indicate first quartiles; horizontal bars within boxes indicate medians; error bars indicate maximums and minimums. Red, northward movement; teal, southward movement; purple, movement that is neither north nor south; dotted arrows, movement that was not observed in the incident-controlled down-sampling because of an insufficient number of sequences. C, D) Movement originating in the eastern and central United States. Only Markov jumps that occurred >2 times are depicted.

According to the 2 incidence-controlled datasets, the MRCA was ≈1997 in both down-sampling exercises (95% HPD 1996.00–1998.52 and 95% HPD 1995.7–1998.25), and the overall mutation rates were estimated to be 4.02 × 10^−4^ and 3.83 × 10^−4^ substitutions/site/year (Table 7; Figure 5). As with the full dataset, the Markov analysis demonstrated that New York and Texas were strong sources of WNV circulation. Significant movement (mean >2 MJ) was detected from Texas to Colorado (20.42 and 20.44 MJ); Texas to New York (12.36 and 11.77 MJ); Texas to Georgia (8.28 and 9.55 MJ); Texas to Virginia (7.14 and 7.732 MJ); New York to Georgia (6.1 and 5.38 MJ); New York to Virginia (4.95 and 3.65 MJ); New York to Colorado (4.04 and 2.66 MJ); New York to Texas (2.66 and 2.73 MJ); Virginia to Georgia (1.55 and 3.62 MJ); and, in dataset 2 only, Virginia to Colorado (1.31 MJ) (Figure 6).

**Table 7:** Statistical support for the incidence-controlled phylogenies determined in study of terrestrial bird migration and West Nile virus circulation, United States*

Variable	Dataset 1				Dataset 2			
	Mean	ESS	95% HPD interval	Mean		ESS	95% HPD interval
Posterior	−36338.60	1385	−36394.36 to −36280.37	−36798.90		1803	−36853.19 to −36740.97
Prior	−3141.96	1187	−3189.54 to −3091.38	−3153.08		1387	−3201.84 to −3104.50
Likelihood	−33196.64	1367	−33229.23 to −33165.79	−33645.82		2115	−33678.80 to −33614.39
MRCA	11.66	4720	10.47 to 12.10	11.97		6985	10.75 to 13.30
UCLD.mean	4.02 × 10^−4^	2336	3.53 × 10^−4^ to 4.55 × 10^−4^	3.83 × 10^−4^		1749	3.31 × 10^−4^ to 4.39 × 10^−4^

*ESS, effective sample size; HPD, highest posterior density; MRCA, most recent common ancestor (years before 2009); UCLD.mean, evolution rate inferred with an uncorrelated clock model with lognormal distribution.

**Figure 7:**
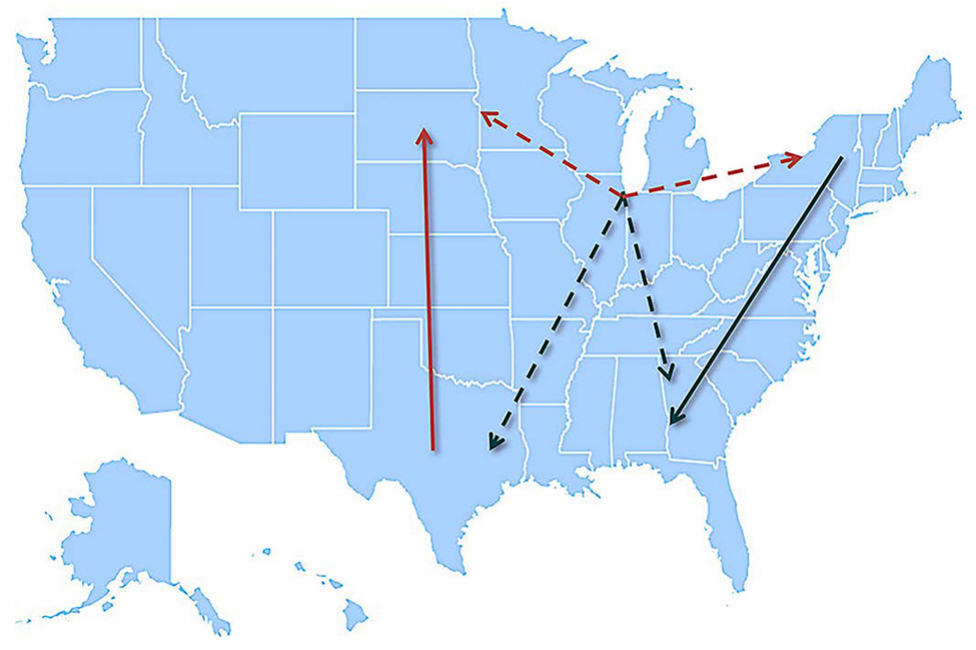
Model summarizing the general patterns of West Nile virus movement in the United States. Red, northward movement; teal, southward movement; dotted arrows, relationships that could not be confirmed in incident-controlled datasets because of an insufficient number of sequences.

Together, the MJ analyses of the incidence-controlled dataset and the full dataset illustrate a consistent pattern of WNV circulation. All southward movement originated in the eastern United States (New York and Virginia), and most of the northward movement originated in the central United States (Texas) (Figure 7).

## Discussion

In recent years, emerging zoonotic diseases caused by Ebola, Zika, Nipah, Middle Eastern respiratory syndrome, and influenza A viruses have become major public health problems, devastating communities and costing millions for public health interventions. Decisive, evidence-based approaches are critical for managing emerging infectious diseases, but effective and efficient countermeasures will be possible only after the relationships between pathogens and their hosts have been thoroughly characterized.

Bird migration has been implicated in the movement of a variety of pathogens (*1*). In particular, characterization of the relationship between avian influenza virus movement and waterfowl migration has supported surveillance and early warning programs (*1*,*43*). However, studies of avian hosts in the Americas have mainly concentrated on the migration of waterfowl to the exclusion of terrestrial birds because waterfowl are easily tracked with banding; thus, their migration has been thoroughly characterized.

The introduction and subsequent spread of WNV into the Americas underscores the invasive potential of emerging pathogens in the New World, as has been recently exemplified by Zika virus, another mosquitoborne flavivirus. Dramatic variations in the location, timing, and intensity of WNV strain collection and sequencing has left the field with a limited understanding of virus circulation patterns and no reliable way of predicting the geographic spread of WNV outbreaks. We have addressed this knowledge gap by characterizing the movement of WNV with regard to the migratory patterns of its natural hosts, terrestrial birds. We compiled 379 virus sequences for analysis, including 289 previously reported sequences from New York, Virginia, Georgia, Illinois, North Dakota, South Dakota, Texas, and Colorado, plus 90 novel sequences from Virginia, Georgia, and Colorado.

Phylogeographic analysis revealed that 3 locations— New York, Illinois, and Texas—accounted for 88.5% of the total WNV MJ inferred. Because New York is the presumed original introduction point for WNV into the United States, its role as a major source of WNV movement was expected. However, 74.2% of the observed MJ originated in Illinois and Texas only. Of note, North Dakota and South Dakota, which are 2 of the states with the highest annual WNND incidence, seem to be strong sinks for WNV moving out of Illinois and Texas.

The contributions of Illinois and Texas to WNV circulation are not surprising because both locations are situated at major convergence points between the Eastern and Central flyways. In the case of Texas, birds from both flyways may avoid long-distance flights across the Gulf of Mexico by traveling along the circa-Gulf route that follows the Gulf Coast through Texas into Mexico. In the case of Illinois, seasonal shifts in terrestrial bird migration routes ensure that Illinois supports birds from the Eastern and Central flyways during annual migrations.

Of note, although mosquito and WNV activity occurs earlier in the southern than in the northern United States, southward migration was detected along the East Coast during our sampling period, 2002–2009, indicating that the southeastern United States is probably a dead end for WNV circulation. Indeed, low-level transmission probably occurs during the winter in warmer locations such as Florida, Texas, and Louisiana. This possibility is supported by isolations of WNV from mosquitoes and birds during December and January in Harris County, Texas (*44*), and suggests that ecologic factors not related to mosquito abundance and WNV activity drive WNV movement along the East Coast. Instead, movement of WNV into the northeastern United States (New York) from Illinois and Texas was observed (in the incident-controlled analysis). These results suggest that introduction of WNV into the northeastern United States originated from the central United States.

Overall, we have defined the pattern of WNV circulation in the United States (Figure 7) and demonstrated looped virus movement patterns in the Eastern and Central flyways that are bridged by Illinois, a region shared between the 2 flyways. This specific pattern correlates with the looped migration patterns of terrestrial birds. Although other geographic regions may contribute to virus movement, there were insufficient virus sequences available from other states to incorporate into this analysis. Thus, on the basis of available information, 3 of the 8 locations considered (New York, Illinois, and Texas) seem to be the preferred sites for efficiently monitoring ongoing WNV evolution.

As new WNV sequences become available, similar phylogeographic methods can be used to develop more detailed information about WNV circulation in the United States. For example, on the East Coast, WNV circulation occurs southward, so surveillance efforts in the Northeast are likely to be more informative than surveillance in the Southeast. Conversely, WNV in the central United States travels northward, so surveillance in the south-central United States is more likely than surveillance in the north-central United States to be informative. Last, the region of overlap between the Eastern and Central flyways is the most likely location for deriving surveillance information because WNV in this area travels in multiple directions.

Collectively, the results of this study illustrate the value of using multidisciplinary approaches to surveillance of infectious diseases, especially zoonotic diseases. Animal migration is shaped by a delicate balance of ecologic factors and anthropomorphic barriers. Natural and manmade events (e.g., climate change, atmospheric fluctuations, habitat destruction) can drastically alter host behavior, which in turn affects the circulation patterns of infectious agents such as WNV. In this study, we defined the patterns of WNV circulation and key areas for surveillance and correlated them with the migratory patterns of their primary reservoir, terrestrial birds. Although this information does not enable prediction of the size of annual WNV outbreaks, these advancements support the construction of targeted surveillance and vector mitigation strategies to predict the annual flow of WNV strains and to enable public health officials to anticipate changes in WNV circulation resulting from altered bird migration.
